# Comparison of the Anesthetic Effects of Alfaxalone Combined with Xylazine or Dexmedetomidine in Captive Formosa Serows (*Capricornis swinhoei*)

**DOI:** 10.3390/ani15030307

**Published:** 2025-01-22

**Authors:** Li-Jen Chang, Toshitsugu Ishihara, Chen-Yeh Lien, Kuan-Sheng Chen

**Affiliations:** 1 Department of Small Animal Clinical Science, Virginia-Maryland College of Veterinary Medicine, Blacksburg, VA 24061, USA; ljchang@vt.edu (L.-J.C.); toshi37@vt.edu (T.I.); 2 Animal Medical Center, Taipei Zoo, Taipei 11656, Taiwan; 3 Department of Veterinary Medicine, College of Veterinary Medicine, National Chung Hsing University, Taichung 40227, Taiwan; 4 Veterinary Medical Teaching Hospital, College of Veterinary Medicine, National Chung Hsing University, Taichung 40227, Taiwan

**Keywords:** alfaxalone, anesthesia, Formosan serow (*Capricornis swinhoei*), hypoventilation, hypoxemia

## Abstract

Formosan serows are an endemic species in Taiwan. Alfaxalone, a γ-aminobutyric acid_A_ agonist, induces or maintains anesthesia in various veterinary species with reported potential adverse effects of respiratory depression and tachycardia. α2-Adrenoceptor agonists exert sedative and muscle relaxation effects, along with substantial cardiovascular adverse effects. Here, we aimed to evaluate the anesthetic effects of alfaxalone combined with xylazine or dexmedetomidine (AX vs. AD, respectively) in Formosan serows. In this randomized, masked study, AX was administered to four serows, and AD was administered to five serows intramuscularly via blow dart. The time and score of induction and recovery were recorded. Post-intubation, isoflurane was administered for maintenance anesthesia. Heart rate (HR), respiratory rate (RR), peripheral saturation of oxygenation (SpO_2_), rectal temperature (RT), and end-tidal CO_2_ (EtCO_2_) were recorded every five to eight minutes. Atipamezole and tolazoline were administered to antagonize dexmedetomidine and xylazine post-procedure, respectively. Both combinations allowed smooth induction and recovery. The AD group exhibited significantly lower HR and SpO_2_ and significantly higher RT and EtCO_2_ than the AX group (both *p* < 0.01). The AD-treated serows exhibited notable muscle rigidity after induction and significant hypoventilation and hypoxemia during the procedure. Although alfaxalone combined with dexmedetomidine or xylazine can produce satisfactory induction and recovery in Formosa serows, notable hypoxemia and hypoventilation are induced by the alfaxalone–dexmedetomidine combination compared to the alfaxalone–xylazine combination.

## Simple Summary

Formosan serow is a wild goat found only in the mountains of Taiwan. Knowledge regarding using drugs for anesthesia in these species is scant. In this randomized, blinded study, we examined the anesthetic effects of two different drug combinations (i.e., alfaxalone–xylazine and alfaxalone–dexmedetomidine) in nine adult Formosan serows and assessed the impact on cardiovascular and respiratory functions. Both combinations ensure anesthesia in 16 min and a smooth recovery within four minutes after administration of antagonists. However, the alfaxalone–dexmedetomidine combination led to low oxygen saturation and high CO_2_ concentration due to depression of respiratory function. In summary, both combinations provide satisfactory anesthesia and recovery; however, the administration of alfaxalone–dexmedetomidine induced significant respiratory depression in anesthetized serows.

## 1. Introduction

Formosan serows (*Capricornis swinhoei*) were the only wild endemic Bovidae in the mountainous regions of Taiwan. Although listed as a least-concern species by the International Union for Conservation of Nature (IUCN), it is a “precious and rare species” protected under the Wildlife Conservation Act of Taiwan because of habitat loss and illegal hunting [1]. Formosan serows were prone to stress, a pathological response triggered by excessive physiological changes, which may result in detrimental consequences [2]. Therefore, appropriate sedation or anesthesia protocols should be carefully considered in Formosan serows undergoing medical procedures to avoid stress-induced complications.

Alfaxalone with 2-hydroxypropyl-β-cyclodextrin has been used extensively in veterinary medicine for a variety of species [3,4]. Alfaxalone is a γ-aminobutyric acid_A_ (GABA_A_) receptor agonist that regulates chloride transport, resulting in central nervous system depression [5]. Alfaxalone-based combinations were shown to induce anesthesia in goats [6,7,8], indicating that alfaxalone is an option for anesthesia induction in ruminants. Moreover, alfaxalone reportedly exerts clinical effects as a satisfactory alternative anesthetic for ketamine for short procedures due to its shorter duration of action compared to ketamine [9]. Hence, evaluating the clinical effects of anesthesia on captive wild ruminants is worth evaluating. However, a notable complication of intramuscular alfaxalone injection in larger species is pain and discomfort owing to its greater injection volume than that of ketamine-based combinations [10]. Therefore, the practicality of alfaxalone-based combinations for remote drug delivery techniques needs to be evaluated.

α2-Adrenoceptor agonists are commonly used sedatives in veterinary medicine and have been applied extensively across diverse species [11]. However, α2-adrenoceptor agonists, such as xylazine, can induce severe respiratory depression and hypoxemia in small ruminants, particularly sheep and goats [12]. Xylazine can reportedly induce alveolar microvascular epithelial cell damage in these species, leading to alveolar rupture and hemorrhage and resulting in pulmonary edema and hypoxemia [13]. Dexmedetomidine has been found to be a more potent α2-adrenoceptor agonist compared to xylazine because of its high α2-adrenoceptor, resulting in better desired clinical effects and undesired adverse effects. Although a decrease in respiratory rate after administration of dexmedetomidine to dogs has been reported [14], the respiratory effects of dexmedetomidine in ruminants have not been well discussed. Therefore, the use of potent α2 adrenoceptor agonists, such as dexmedetomidine, for anesthesia induction in Formosan serows anesthesia warrants further investigation.

The objective of the current study was to compare the anesthetic and cardiopulmonary effects of alfaxalone combined with xylazine or dexmedetomidine in captive Formosan serows. We hypothesized that the alfaxalone–dexmedetomidine (AD) combination may elicit a superior anesthetic effect than the alfaxalone–xylazine (AX) combination; however, severe respiratory depression could occur with the use of the alfaxalone–dexmedetomidine combination.

## 2. Materials and Methods

### 2.1. Animals

This randomized, masked clinical study included nine Formosan serows (four males and five females) kept in captivity at Taipei Zoo, Taipei, Taiwan (24°59′42″ N, 121°35′3″ E). All included animals had undergone annual health examinations. The physiological status of each subject was evaluated based on the American Society of Anesthesiologists Physical Status (ASA-PS) classification system. All subjects were assigned to ASA-PS I or II, which is associated with lower anesthetic risks [15]. Food was withheld for at least 12 h before anesthesia. The study protocol was approved by the Institutional Animal Care and Use Committee of the Taipei Zoo (protocol code 10502).

The anesthesia protocol was assigned randomly using a lottery system on the day of the procedure. Alfaxalone–dexmedetomidine (Dexdomitor^®^; Zoetis, Taipei, Taiwan) was administered to five serows, and alfaxalone (Alfaxan^®^; E-Rei, Taipei, Taiwan)–xylazine (Balazine^®^ 10%; Health-tech Pharmaceutical, Taipei, Taiwan) was administered to four serows from January to July 2016. Table 1 presents the demographic characteristics of the included animals. The serows were aged between 6 and 15 years and had an average body weight of 21.08 ± 4.35 kg.

### 2.2. Anesthesia

Both combinations were loaded in two 5 mL syringes and administered intramuscularly via a blow dart by two well-trained and experienced veterinarians. The anecdotal doses of xylazine and dexmedetomidine used at Taipei Zoo were 2–3 mg/kg and 10–20 µg/kg, respectively. A pilot study determined the alfaxalone dosage as 3–4 mg/kg. Precise body weights were measured after anesthesia induction. Induction time was recorded and defined as the duration between administering the combination to the animal presenting lateral recumbency. Induction quality was assessed and recorded by a blinded observer using the scoring system detailed in Table 2. Upon complete induction, the animal was relocated to the animal medical center of Taipei Zoo for endotracheal intubation and intravenous catheterization. The distance from the enclosure to the animal medical center was approximately 2 km, necessitating approximately 5–10 min for transportation. Oxygen (10 L/min) was administered via an insufflation tube in one side of the serow’s nostrils during transportation, with the neck elevated using a towel to maintain the mouth and nostrils downward to minimize the risk of aspiration. The serows’ trachea was intubated with 5–7 mm (I.D.) endotracheal tubes (Medline, Fort Pierce, FL, USA) depending on body weight. If the first intubation attempt failed, 5% of isoflurane (vaporizer setting) was administered via a mask to facilitate intubation. An intravenous catheter was placed on either side of the cephalic vein with a 20 G IV catheter (Terumo, Taipei, Taiwan). Anesthesia was maintained using isoflurane in a rebreathing circuit. The oxygen flow rate was 20–40 mL/kg/min. Isoflurane and oxygen were delivered using Dayex-Ohmeda Excel 210SE^®^ (Ohmeda, Madison, WI, USA). Animals breathed spontaneously throughout the procedure.

### 2.3. Assessment of Physiological Parameters and Recovery

**Figure 1:**
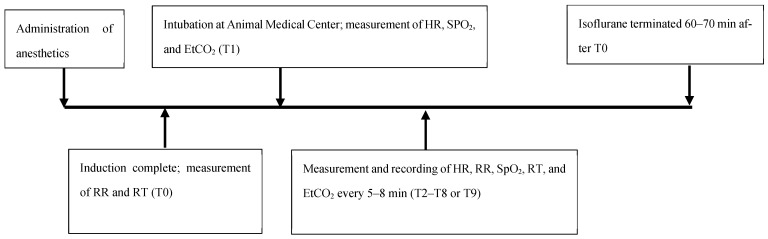
Timeline illustration of this study. The time points and associated events are shown. The measurement of physiological parameters was performed every 5–8 min after the first measurement of HR, SpO_2_, and EtCO_2_. EtCO_2_, end-tidal CO_2_; HR, heart rate; RR, respiratory rate; RT, rectal temperature; SpO_2_, peripheral saturation of oxygenation.

Heart rate (HR), respiratory rate (RR), peripheral saturation of oxygenation (SpO_2_), rectal temperature (RT), and end-tidal CO_2_ (EtCO_2_) were monitored and recorded every 5–8 min during the procedure. The duration of anesthesia was approximately 60–70 min. RR and RT were recorded after induction (time 0). Time 1 was marked as the time immediately after intubation when recording other physiological parameters was initiated, with subsequent time points at 5–8 min intervals during the procedure. The heart rate was measured by auscultation and recorded. The respiratory rate was counted and recorded by observing the movement of the reservoir bag attached to the anesthesia machine. Peripheral pulse oxygen saturation was measured using the Masimo Rad-5^®^ pulse oximeter (Masimo, Irvine, CA, USA), and the probe was placed on the tongue; the tongue was covered by a saline-rinsed 4 × 4 gauze. End-tidal CO_2_ was measured using EMMA^®^ capnography (Masimo, Irvine, CA, USA). The rectal temperature was measured using a veterinary digital thermometer (Vet thermometer^®^, Shandong, China). Hypoxemia was defined as SpO_2_ < 90% [16]. If the first measured RT was below 37.5 °C, the serows were warmed with a circulating water heating pad (Gaymar Stryker^®^ TP 650, Orchard Park, NY, USA). Isoflurane was discontinued upon completion of the procedure, and the recording was ceased. The timeline of this study is shown in Figure 1.

**Figure 2:**
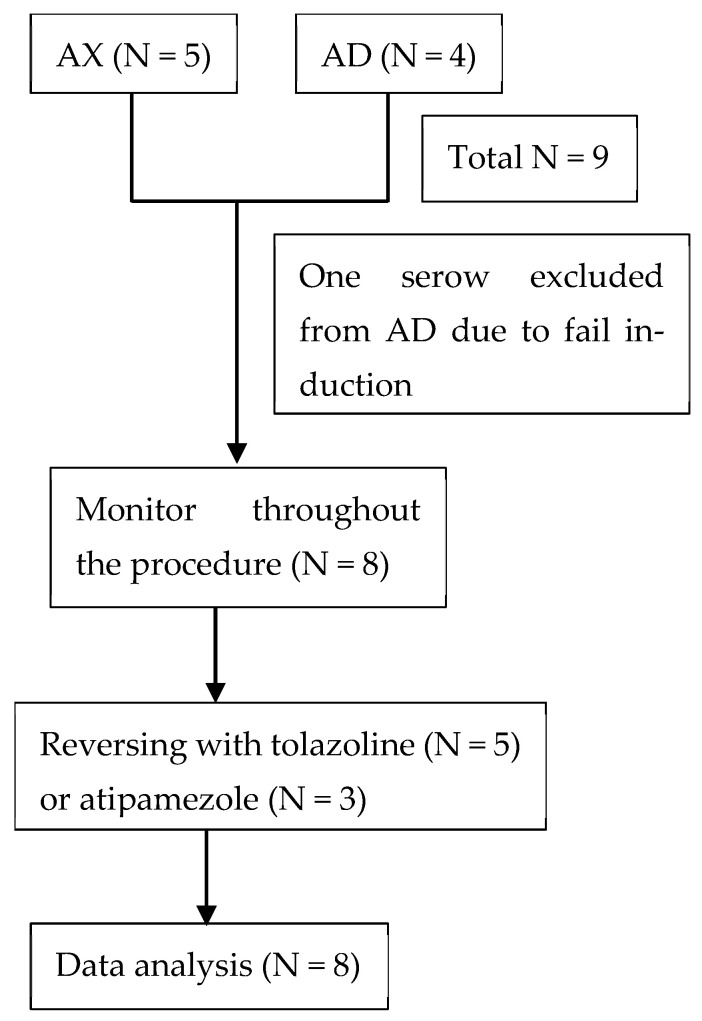
Consort diagram for study design. AX, alfaxalone–xylazine; AD, alfaxalone–dexmedetomidine.

Subsequently, tolazoline (4 mg/kg, self-compounded) and atipamezole (same volume as dexmedetomidine; Antiseden^®^; Zoetis, Taipei, Taiwan) were administered to serows intravenously to reverse the effects of xylazine and dexmedetomidine, respectively, when relocated to the enclosure, which was about 10–15 min after turning off isoflurane. Recovery time was defined as the period between the administration of antagonists and the animal’s first attempt to stand. A blinded observer assessed and recorded recovery quality using the criteria listed in Table 1. The anesthetic scoring system was adapted from a previous study [6]. The consort diagram to illustrate the study design is presented in Figure 2.

### 2.4. Statistical Analysis

Normal probability plots were inspected to assess data distribution, and a normal distribution of the data was confirmed. The dosages of alfaxalone, xylazine, dexmedetomidine, and atipamezole were calculated based on the measured body weight of animals. The results are presented as mean ± standard deviation (SD) or median and range, as appropriate. Boxplots with individual data points were used to visualize the distribution of induction time, induction score, recovery time, and recovery score. The Wilcoxon rank-sum test was employed to detect between-group differences. A mixed model analysis of variance was used to determine differences in physiological parameters between the groups. Data analyses were performed using SAS software (Version 9.4, Cary, NC, USA). A *p*-value of <0.05 was deemed statistically significant.

## 3. Results

In the AX group, the average doses of xylazine and alfaxalone were 2.2 ± 0.3 mg/kg and 3.8 ± 0.72 mg/kg, respectively. In the AD group, the average doses of dexmedetomidine, alfaxalone, and atipamezole were 14.5 ± 6.8 µg/kg, 6.12 ± 4.47 mg/kg, and 117.5 ± 85.9 µg/kg, respectively.

In the AD group, one Formosan serow failed to achieve lateral recumbency 30 min after the first intramuscular injection via blow dart. However, following a second intramuscular injection via hand injection, the animal could be approached and blindfolded. A total of 12.8 mg/kg of alfaxalone was administered to this serow and achieved lateral recumbency approximately 40 min after the first injection; however, the serow displayed exaggerated struggling during transportation. We decided to abort the procedure and administered atipamezole to induce recovery. Accordingly, this serow was only included in calculating average drug doses and was excluded from the subsequent statistical analyses. Upon excluding this subject, the average alfaxalone dose was 3.89 ± 0.47 mg/kg for the AD group.

**Figure 3:**
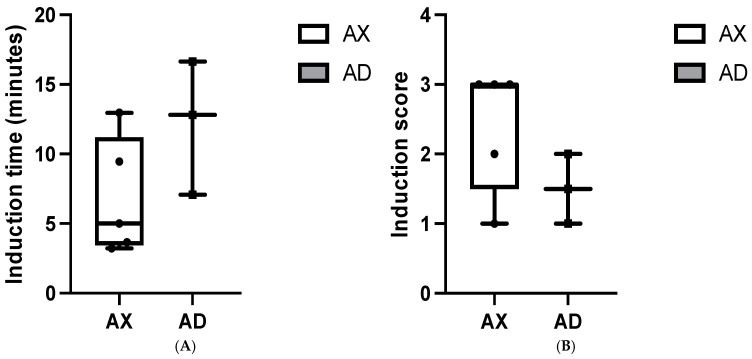

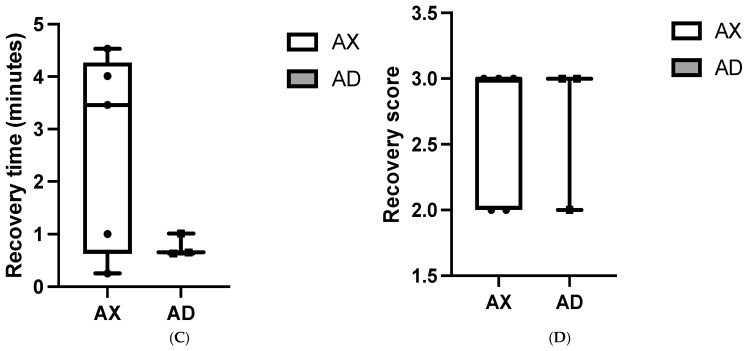
Induction time (**A**), induction score (**B**), recovery time (**C**), and recovery score (**D**) in Formosan serows administered alfaxalone-xylazine (AX) or alfaxalone-dexmedetomidine (AD).

The median induction times were 5 (3.2–12.96) min and 12.48 (7.08–16.65) min in the AX and AD group, respectively. The AX and AD groups had median induction scores of 3.0 (1.0–3.0) and 1.5 (1.0–2.0), respectively. The median recovery times were 3.46 (0.25–4.53) min in the AX group and 0.65 (0.63–1.01) min in the AD group. The AX and AD groups had median recovery quality scores of 3.0 (2.0–3.0) and 3.0 (3.0–3.0), respectively. No significant differences were detected between the two groups regarding induction time, induction score, recovery time, and recovery score (Figure 3A–D). However, serows in the AD group (3/3) exhibited notable excitement during induction. Although lateral recumbency could be observed within approximately 16 min, serows in the AD groups showed paddling and muscle tremors after achieving lateral recumbency. Only one individual in the AX group (1/5) was observed mild muscle tremors during induction. All serows included in this study were successfully intubated without necessitating the use of isoflurane.

**Figure 4:**
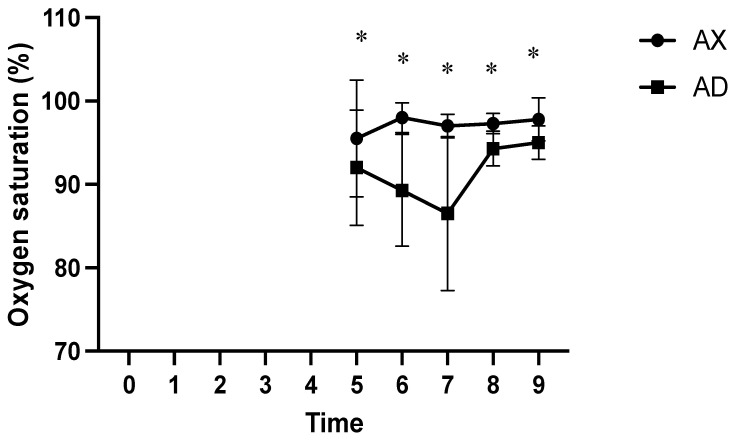
Peripheral oxygen saturation in Formosan serows administered alfaxalone-xylazine (AX) or alfaxalone-dexmedetomidine (AD). Time 0 indicates the moment after induction before intubation. Time 1 indicates the moment immediately after intubation, with subsequent time points set at 5-8 min intervals. Asterisks denote statistical differences between the AX and AD groups: * *p* < 0.05).

**Figure 5:**
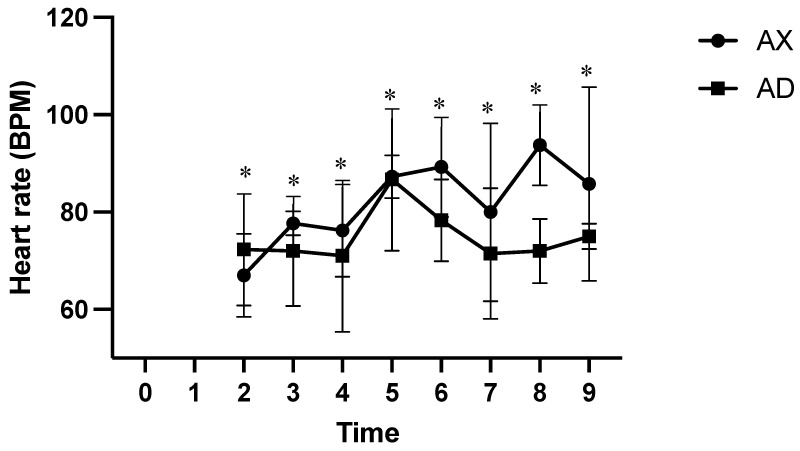
The heart rate of Formosan serows administered alfaxalone-xylazine (AX) or alfaxalone-dexmedetomidine (AD). Time 0 indicates the moment after induction before intubation. Time 1 indicates the moment immediately after intubation, with subsequent time points set at 5-8 min intervals. Asterisks denote statistical differences between the AX and AD groups: * *p* < 0.05. BPM, beats per minute.

**Figure 6:**
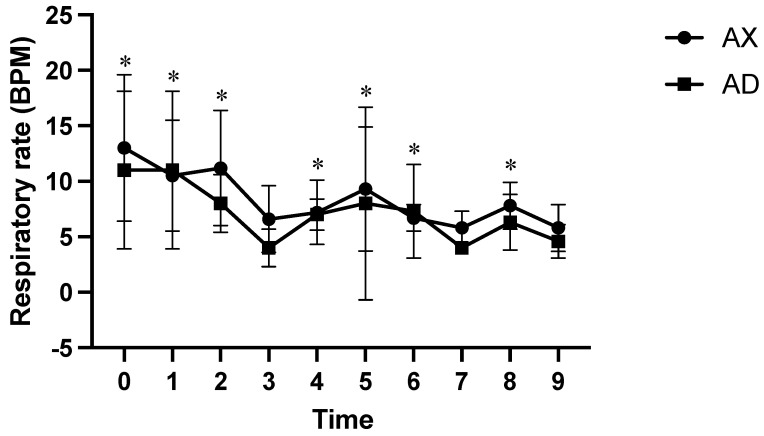
The respiratory rate of Formosan serows administered alfaxaloneu-xylazine (AX) or alfaxalone-dexmedetomidine (AD). Time 0 indicates the moment after induction before intubation. Time 1 indicates the moment immediately after intubation, with subsequent time points set at 5-8 min intervals. Asterisks denote statistical differences between the AX and AD groups: * *p* < 0.05. BPM, beats per minute.

**Figure 7:**
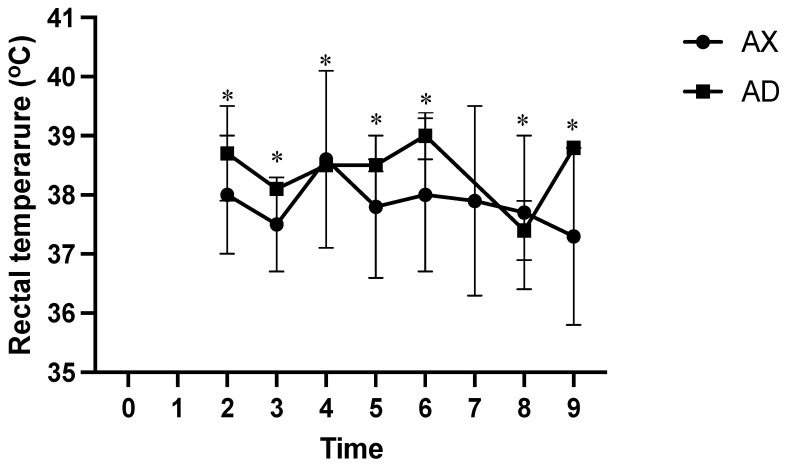
The rectal temperature of Formosan serows administered alfaxalone-xylazine (AX) or alfaxalone-dexmedetomidine (AD). Time 0 indicates the moment after induction before intubation. Time 1 indicates the moment immediately after intubation, with subsequent time points set at 5-8 min intervals. Asterisks denote statistical differences between the AX and AD groups: * *p* < 0.05.

**Figure 8:**
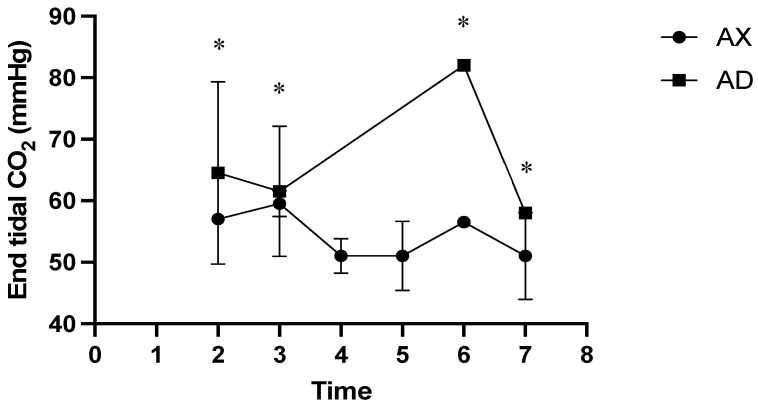
End-tidal CO_2_ of Formosan serows administered alfaxalone-xylazine (AX) or alfaxalone-dexmedetomidine (AD). Time 0 indicates the moment after induction before intubation. Time 1 indicates the moment immediately after intubation, with subsequent time points set at 5-8 min intervals. Asterisks denote statistical differences between the AX and AD groups: * *p* < 0.05.

At all measured time points, the AD group had significantly lower SpO_2_ values than the AX group (*p* < 0.01; Figure 4). At time points 2 and 5, the HR was significantly lower in the AX group than in the AD group (*p* < 0.01; Figure 5), whereas the HR was significantly lower in the AD than in the AX group at all other measured time points (*p* < 0.01). At time points 1, 4, and 6, the AX group had significantly lower RR values than the AD group (*p* < 0.01; Figure 6), whereas the AD group had a significantly lower RR than the AX group at time points 0, 2, and 8 (*p* < 0.01). Although the AD group had lower RR values than the AX group at time points 3, 5, 7, and 9, the difference was non-significant. The AD group exhibited significantly higher RTs than the AX group at all time points except for time points 7 and 8 (*p* < 0.01; Figure 7). Additionally, the AD group had a significantly higher EtCO_2_ than the AX group at all measured time points (*p* < 0.05; Figure 8).

During the procedure, no animals experienced any notable complications, such as rumen bloating, regurgitation, or aspiration pneumonia. Additionally, no animal displayed any sign of dysphoria, salivation, or whining during recovery.

## 4. Discussion

The results of this study indicate that alfaxalone combined with xylazine or dexmedetomidine could induce smooth anesthesia and uneventful recovery in captive Formosan serows, although mild muscle tremors could be observed in those received AD combination. These findings were in line with the conclusions of a previous study [8], which reported that administering 3 mg/kg alfaxalone intravenously could effectively induce anesthesia in goats. The average dose of alfaxalone used in this study was approximately 4 mg/kg higher than that employed in the aforementioned study. Reportedly, a higher alfaxalone dose is required for intramuscular administration [17]; hence, the discrepancy could be attributed to the distinct route of administration.

In the AD group, one serow was excluded from this study owing to induction failure 40 min after the first blow dart and vigorous struggling during transportation. Upon approaching the animal for blindfolding, we found liquid dampening the haircoat surface close to the darting site; hence, incomplete injection was suspected, which may underlie the induction failure. This finding supports the assumption that a large injection volume increases the incidence of injection failure via remote drug delivery systems, leading to induction failure [18].

Although statistically non-significant, the AX combination was associated with a shorter duration of anesthesia onset than the AD combination; however, more paddling and muscle tremors were observed during the induction phase with the AD combination. This finding is consistent with a previous study that observed similar clinical signs when administering alfaxalone combined with dexmedetomidine intramuscularly in pigs [17]. Intramuscular administration of alfaxalone was found to induce muscle tremors and paddling in dogs and cats [19,20,21]. Therefore, combining alfaxalone with other sedatives or muscle relaxants, such as α2 adrenoceptor agonists, benzodiazepines, or opioids, has been recommended to mitigate the adverse effects [19]. Although the average dose of alfaxalone was similar (approximately 4 mg/kg) in both groups, only one serow in the AX group exhibited muscle tremors and paddling during induction. This finding could be explained by the different dosages of the α2 adrenoceptor agonists. The dose of xylazine used in this study was similar to that used in a previous report [22], whereas the dose of dexmedetomidine was lower than a published dosage [23]. Accordingly, alfaxalone should be used concurrently with an appropriate dose of α2 adrenoceptor agonists to minimize the incidence of adverse effects, such as muscle tremors, during induction.

In this study, the AD group had significantly lower SpO_2_ than the AX group at all measured time points, thereby aligning with the findings of previous reports on the use of alfaxalone to induce anesthesia in goats and highlighting the importance of continuous oxygen supplementation during anesthesia [6,7,8]. Alfaxalone reportedly induces post-induction apnea following rapid intravenous injection or substantial respiratory depression at high doses [24,25]; however, alfaxalone does not induce detrimental respiratory depression under clinically relevant doses [26]. In this study, we ensured adequate oxygen supply from induction to endotracheal tube removal. Although oxygen was supplied throughout this study to all serows, those in the AD group continued to exhibit hypoxemia until 40–50 min post-induction. Conversely, serows in the AX group had SpO_2_ values that exceeded 90% throughout thi study, indicating that hypoxemia was primarily due to dexmedetomidine. Furthermore, serows in the AD group exhibited significantly higher ETCO_2_ readings than serows in the AX group throughout the study period, indicating that the serows in the AD group suffered from respiratory depression and hypoventilation. Considering the use of comparable alfaxalone doses between groups, respiratory depression could be attributed to the use of dexmedetomidine. Administration of dexmedetomidine has been shown to result in hypercapnia and hypoxemia [27]. Although the dose of dexmedetomidine used in this study was lower than previously reported doses in captive serows [22,23], the incidence of hypoxemia was more profound than that reported previously. The result of the current study implies that the α2 adrenoceptor agonist-induced hypoxemia and respiratory depression in sheep and goats could be due to individual and breed differences [28]. Nonetheless, dexmedetomidine may result in more severe peripheral vasoconstriction than xylazine, which potentially affects the accuracy of peripheral oxygen saturation monitoring. Therefore, monitoring arterial oxygen tension could provide more accurate evidence of oxygen saturation.

Serows in the AD group had higher HRs than those in the AX group for the first 10 to 15 min after induction, whereas HRs at other time points were significantly lower in the AD group. This finding may reflect the pharmacodynamic profiles of dexmedetomidine, that is, potent α2 adrenoceptor receptor agonists induce a pronounced sympatholytic effect characterized by hypotension and bradycardia 40 to 45 min after administration of dexmedetomidine [29]. Blood pressure was not monitored in this study; however, it is impossible to recognize that bradycardia and hypotension resulted from the sympatholytic effect. Although alfaxalone does not induce notable adverse cardiovascular effects, it may increase HR owing to its chronotropic effect [30]. Therefore, given the comparable alfaxalone dosages in this study, it is reasonable to assume that the use of a potent α2 adrenoceptor receptor agonist resulted in bradycardia. Arterial blood pressure should be monitored in future studies to determine whether changes in HR were due to α2 adrenoceptor receptor agonists.

Additionally, serows in the AD group had substantially higher body temperature than serows in the AX group, except at time point 8. Although the normal body temperature of serows is yet to be reported, it is believed to be comparable to the body temperature of a domestic goat (38.5–39.7 °C). The RT measured in this study remained within the normal range. The increase in RT in the AD group is likely due to the more pronounced muscle tremors and paddling during induction. Intramuscular administration of alfaxalone does not markedly impact body temperature in dogs [31]; however, α2 adrenoceptor agonists could lead to hypothermia due to sympatholytic activity [32]. Therefore, the higher RT in the AD group could be due to muscle excitement during induction. Biomarkers associated with muscle damage, such as alanine transaminase, aspartate transaminase, alkaline phosphatase, creatine kinase, lactate dehydrogenase, and lactate, were not assessed in this study. Hence, it is difficult to conclude that the higher RT observed in the AD group was directly related to the observed muscle excitement.

In the current study, atipamezole and tolazoline were used to reverse the clinical effects of dexmedetomidine and xylazine, respectively. Despite the intravenous administration of antagonists, all serows experienced uneventful and smooth recovery. Serows in the AD group regained consciousness and could stand and walk within 1 min of atipamezole administration. In contrast, serows in the AX group recovered completely within 4 min of tolazoline administration. This finding aligns with those of previous studies [33,34], indicating that appropriate antagonists were selected in this study. Furthermore, intravenous administration of the antagonists did not induce excitement. This finding differs from that of a previous study [23], where the authors found that intravenous administration of atipamezole to serows treated with a tiletamine–zolazepam–dexmedetomidine combination resulted in prolonged and unsatisfactory recovery. The discrepancies between the two studies may be due to the use of distinct anesthetics for induction. It is well known that alfaxalone has a shorter duration of action in goats [8] than tiletamine–zolazepam [35]. Therefore, alfaxalone was already metabolized when antagonists were administered, facilitating a smooth and uneventful recovery. The results of this study are consistent with those of a previous report, where alfaxalone-induced anesthesia elicited smooth and calm recovery in goats [8].

The results of this study revealed that the alfaxalone-based combinations had an injection volume of nearly 10 mL, which is substantially larger than the injection volumes of ketamine- or tiletamine–zolazepam-based combinations reported previously [23]. The occurrence of induction failure provides strong evidence that the injection volume can impact the effectiveness and success rate of remote drug delivery. In the current study, two 5 mL darts were used to deliver the pre-determined combinations to the serow, which complicated the completion of injections because the serow became anxious after the first dart. This finding is in line with that of a previous study, which concluded that excessive injection volumes could raise the incidence of remote drug delivery failures [18]. High-concentration alfaxalone (4%) has been successfully applied to zoo species with remote drug-delivery systems [36]. Based on the findings of the current study, serows require an intramuscular injection volume of 0.4 mL/kg (4 mg/kg) of 1% alfaxalone for induction, which exceeds the recommended intramuscular injection volume (0.05–0.25 mL/kg) [37]. However, when using 4% alfaxalone, the lower injection volume (0.1 mL/kg) adheres to the recommended injection volume and minimizes pain upon injection due to excessive injection volumes [10].

This study has several limitations. First, we did not set up an arterial line to monitor arterial blood pressure, arterial oxygen tension, and arterial carbon dioxide tension. Therefore, effectively assessing oxygenation and hemodynamics was challenging. However, EtCO_2_ was monitored using capnography, making the assessment of respiratory function accurate. Second, time and climate effects could be potential confounding factors owing to the prolonged study duration (six months) and weather changes during the study period. In 2016, the recorded environmental temperatures in Taipei City were 16.9 °C during winter and 28.2 °C during summer. Climatic changes can influence the quality of anesthesia and result in differences in the efficacy of anesthetics [38]. Finally, only eight Formosan serows (one in the AD was excluded due to induction failure) were included in this study, resulting in a small sample size. Several non-significant results suggest that this study may lack sufficient power to detect meaningful differences in certain outcomes, which could be a practical limitation in the study of endemic wildlife.

## 5. Conclusions

In conclusion, alfaxalone combined with xylazine or dexmedetomidine could induce anesthesia within 16 min in captive Formosan serows. Formosan serows could stand up and walk steadily within 4 min of intravenous atipamezole or tolazoline administration; however, alfaxalone combined with dexmedetomidine resulted in profound respiratory depression and hypoxemia in captive Formosan serows. Therefore, oxygen supplementation is necessary when using the AD combination for anesthesia induction in serows. Furthermore, alfaxalone-based combinations require greater injection volume than ketamine or tiletamine–zolazepam-based combinations, leading to an increased incidence of remote drug delivery failure.

## Figures and Tables

**Table 1:** Demographics of Formosan serows in this study.

	Sex	Age (yrs)	Body Weight (kg)
AX	F	9	23.1
	F	10	23.1
	F	6	16.3
	MC	11	23.4
	MC	15	21.8
AD	MC	13	22.5
	F	12	23.4
	MC	10	25.4
	F	14	23.1

AX, alfaxalone–xylazine; AD, alfaxalone–dexmedetomidine; MC, male castrated; F, female.

**Table 2:** Anesthetic quality scoring system chart.

Score	Induction	Recovery
0	High excitement (vocalizes, jumps, or attempts to escape during physical restraint, unable to place the endotracheal tube)	Poor (several uncoordinated attempts to stand, ataxic)
1	Moderate excitement (some struggling, falling, or slipping after becoming recumbent; able to place the endotracheal tube)	Relatively Poor (several coordinated attempts to stand, ataxic)
2	Low excitement (some struggling, no falling or slipping; may or may not be intubated within 60 s)	Relatively calm (1–2 coordinated attempts to stand with minimal ataxia)
3	Excitement-free induction (no outward signs of excitement, tracheal intubation easy)	Excitement-free recovery (no outward signs of excitement, stood up smoothly and quickly)

## Data Availability

The raw data supporting the conclusions of this study will be made available by the authors upon request.

## Abbreviations

The following abbreviations are used in this manuscript:

HR	Heart rate
RR	Respiratory rate
EtCO_2_	End-tidal CO_2_
SpO_2_	Peripheral saturation of oxygenation
RT	Rectal temperature
AX	Alfaxalone–xylazine
AD	Alfaxalone–dexmedetomidine
